# Pancreatic Cancer in the Holobiont and Therapeutic Targets: A Review

**DOI:** 10.3390/jcm15093225

**Published:** 2026-04-23

**Authors:** Charlotte Terry, Lewis A. Hall, James Halle-Smith, Lindsey A. Edwards, Shivan Sivakumar, Iain Chapple, Andrew Beggs, Tariq Iqbal, Keith J. Roberts

**Affiliations:** 1 Department of HPB and Liver Transplantation, Queen Elizabeth Hospital Birmingham, University Hospitals Birmingham NHS Trust, Birmingham, B15 2GW, UK; charlotte.terry2@nhs.net (C.T.); lewis.hall6@nhs.net (L.A.H.); james.halle-smith@uhb.nhs.uk (J.H.-S.); s.sivakumar@bham.ac.uk (S.S.); 2 College of Medical and Health Science, University of Birmingham, Birmingham B15 2TT, UK; i.l.c.chapple@bham.ac.uk (I.C.); andrew.beggs@uhb.nhs.uk (A.B.); t.h.iqbal@bham.ac.uk (T.I.); 3 Centre for Host Microbiome Interactions, King’s College London, London WC2R 2LS, UK; lindsey.edwards@kcl.ac.uk; 4 Birmingham NIHR Biomedical Research Centre in Inflammation, University of Birmingham, Birmingham B15 2TT, UK; 5 Department of Gastroenterology, University Hospitals Birmingham NHS Trust, Birmingham B15 2GW, UK

**Keywords:** pancreatic ductal adenocarcinoma (pancreatic cancer), gut microbiome, oral microbiome, shotgun metagenomics, microbial diversity, functional microbial pathways, chemotherapy response

## Abstract

Increasing evidence suggests pancreatic cancer develops within a host–microbe ecosystem in which microbial communities across anatomical niches interact with tumour biology, immune regulation, metabolism, and therapeutic response. This review examines pancreatic cancer through the lens of humans as holobionts, integrating evidence from the oral, gut, biliary, and intratumoural microbiomes. Epidemiological and sequencing studies demonstrate consistent microbial alterations across these niches in pancreatic cancer, including oral dysbiosis associated with periodontal pathogens, gut microbial shifts toward pro-inflammatory taxa, disease-specific biliary microbial signatures, and the presence of distinct intratumoural microbial communities. Mechanistic studies indicate that intestinal barrier disruption, microbial translocation, immune and metabolite signalling can influence tumour immune architecture, macrophage polarisation, T-cell infiltration, oncogenic signalling pathways, and chemotherapeutic metabolism, particularly inactivation by tumour-associated bacteria. Microbiome-driven shifts in immunometabolism can reprogramme immune-cell metabolic pathways, impairing effective T-cell activation, promoting tumour-supportive macrophage phenotypes. Emerging therapeutic strategies aim to modulate the microbiome–tumour axis, including dietary interventions, probiotics and immunonutrition, faecal microbiota transplantation, engineered microbial therapies, and microbiome-informed antibiotic strategies. While pre-clinical findings are compelling and early-phase clinical studies suggest feasibility, most evidence remains associative and heterogeneous across cohorts and methodologies. Understanding pancreatic cancer as a multi-site ecological system may help explain inter-patient variability in disease progression and treatment response. This could usher in a new era for therapeutic manipulation where future progress will depend on longitudinal, multi-omic, and interventional studies to determine whether microbiome-targeted strategies can produce clinically meaningful improvements in pancreatic cancer outcomes.

## 1. Introduction

Pancreatic ductal adenocarcinoma (PDAC) remains one of the most lethal malignancies worldwide, with approximately 5% five-year survival globally [1,2,3]. Most patients are incurable at diagnosis, and the majority of those who receive potentially curative treatment (surgery and chemotherapy) die of cancer recurrence. Despite centralisation of services, advances in surgical technique, improved peri-operative care, and the evolution of systemic therapies, survival gains remain modest [4,5,6,7], and the lack of early diagnostic strategies, treatment resistance, and treatment-related toxicity continue to limit therapeutic success [8]. For simplicity, henceforth in this review, ‘pancreatic cancer’ refers to its most common subtype: PDAC.

Outcomes vary between populations, suggesting that environmental and lifestyle factors contribute to disease progression, suggesting the importance of host–environment interactions in pancreatic cancer biology [5].

The “holobiont” describes humans as superorganisms [9,10,11], comprising 50% human and 50% bacterial cells and living in harmony with our symbiotic microbial partners in health [12]. Within this framework, a tumour microbiome interacts with the oral, gut, and biliary microbiomes, together with systemic immune responses, to form an integrated host-microbe unit where prevention of cancer requires a paradigm shift in thinking that embraces the holobiont as one entity rather than distinct microbial and host compartments (Kilian et al., 2016 [12]). Increasing attention has turned to the human microbiome as a regulator of immune function, metabolism, and tumour biology. Rather than acting independently, microbial communities across anatomical sites interact with host physiology as part of a composite biological system. Understanding pancreatic cancer through the lens of the human holobiont and adopting a multi-site perspective may help explain heterogeneity in tumour progression and therapeutic response [13,14].

The tumour microenvironment is central to pancreatic cancer development. A dense stromal compartment occupies the majority of the tumour mass and contains substantial immune cell populations [15]. This stroma is dynamic and shapes tumour growth and metastatic potential through the establishment of an immunosuppressive milieu. Experimental evidence suggests that intratumoural microbial communities may contribute to this immune architecture. In murine models, differential activation of Toll-like receptor signalling in monocytic cells has been associated with reduced M1 macrophage polarisation and diminished CD8^+^ T-cell infiltration [16], implicating the tumour microbiome in modulation of local immune tone. Emerging evidence further shows that microbiome-driven shifts in immunometabolism can reprogramme immune-cell metabolic pathways, impairing effective T-cell activation, promoting tumour-supportive macrophage phenotypes [17].

The microbiota, their metabolic capability or respective metabolites; therefore, represent a potentially modifiable component of this system. Microbial manipulation has demonstrated therapeutic relevance in other malignancies, particularly in modulating responses to immunotherapy [8,18]. In pancreatic cancer, microbial alterations have also been identified in early disease states and may hold value as biomarkers of risk or early detection [19,20].

Interpreting microbiome studies in PDAC is complicated by considerable variability between cohorts, which is not consistently clear across the literature. Several methodological and clinical factors contribute to this, including differences in sequencing technique, sampling site, treatment status, disease stage, comorbidities, and prior antibiotic exposure. Broader influences such as geography and diet further shape baseline microbiota and may contribute to population-specific differences. These sources of variability make direct comparison between studies difficult and may explain inconsistencies in reported microbial associations with PDAC.

With these limitations in mind, the aim of this review is to examine pancreatic cancer within the framework of the human holobiont. Synthesising evidence across the oral, biliary, tumour, and gut microbiomes and evaluating emerging microbiome-targeted interventions and their potential to influence outcomes in a dismal disease, the review aims to highlight consistent findings where they exist and acknowledge inter-cohort heterogeneity where they do not.

The literature search was performed in February 2026, using PubMed, Embase, and OpenEvidence platforms. Key search terms included “pancreatic cancer”, “microbiome”, “holobiont”, “oral microbiome”, “biliary microbiome”, “tumour microbiome”, “faecal microbiome”, and “faecal microbiota transplantation”. Reference lists of included articles were manually scrutinised to identify additional relevant literature.

## 2. The Holobiont

### 2.1. The Oral Microbiome

Accumulating epidemiological evidence links periodontitis and edentulism with pancreatic cancer, with periodontitis identified as an independent risk factor for pancreatic cancer [21,22]. The most widely recognised periodontal pathogens associated with advanced disease are Gram-negative obligate anaerobes, with some facultative anaerobes in early disease stages [23], supporting a potential role for chronic oral inflammation and microbial exposure in carcinogenic pathways. Multiple studies have compared the oral microbiome of patients with pancreatic cancer to healthy controls, and in some cases to patients with benign pancreatic disease [19,24,25,26]. Across cohorts, reduced alpha diversity has been reported in pancreatic cancer compared with healthy controls and benign pancreatic disease groups [27]. Sun et al. further demonstrated altered species uniformity using the Heip index, with the pancreatic cancer group exhibiting higher evenness compared with benign pancreatic disease and healthy control groups, indicating compositional restructuring rather than simple species loss [27].

Alterations in oral microbial composition have also been detected in early-stage pancreatic cancer, with the study authors suggesting this as a basis for the early detection of pancreatic cancer [19]. However, the utility of such an analysis in routine clinical practice is likely to provide very low specificity, and oral microbial communities also vary substantially by sampling site. Saliva and tongue samples generally demonstrate higher alpha diversity than buccal or gingival sites, and beta-diversity analyses confirm distinct clustering between anatomical locations [28]. Standardisation of sampling methodology is therefore essential when interpreting and comparing oral microbiome studies.

**Figure 1:**
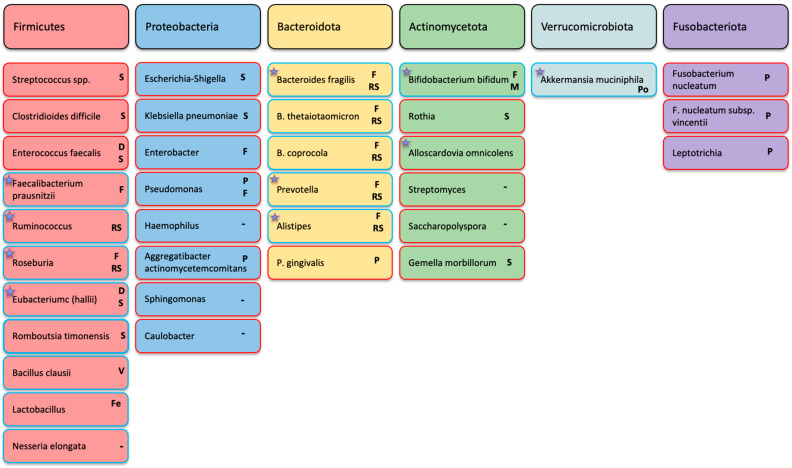
Taxa associated with pancreatic cancer within the holobiont: Overview of bacterial taxa reported in association with pancreatic ductal adenocarcinoma (PDAC), grouped by major phyla. Proteobacteria (e.g., *Escherichia*, *Klebsiella*, *Pseudomonas*) are frequently enriched in tumour and biliary samples, whereas short-chain fatty acid–producing Firmicutes (e.g., *Faecalibacterium*, *Roseburia*, *Ruminococcus*) are commonly depleted in faecal cohorts. Oral-associated taxa, including *Porphyromonas gingivalis* and *Fusobacterium nucleatum*, have been linked to PDAC risk and variably detected across compartments. Those bacteria generally associated with better outcomes are highlighted in blue and those generally associated with worse outcomes are in red. However, patterns differ by sampling site and methodology; most associations remain observational. ☆ Indicates bacteria that have a role in short-chain fatty acid production either directly or indirectly. Main bacterial food source is in bold (S—simple carbohydrates, F—fibre-rich food, RS—resistant starch, V—various carbohydrates, Fe—fermented dairy/food, P—protein-rich food, D—dairy, Po—polyphenol-rich food).

At the taxonomic level, enrichment of species within the phylum Firmicutes and *Prevotella* spp. has been observed in pancreatic cancer (Figure 1) [19]. Other studies have identified Prevotellaceae as dominant without demonstrating clear inter-group differences, highlighting cohort variability [27]. Associations with *Fusobacterium* are similarly heterogeneous, with enrichment being reported in some analyses, whereas prospective data suggest a modest inverse association with pancreatic cancer risk (OR 0.94) [26,29,30]. *Leptotrichia* has also been associated with reduced pancreatic cancer risk (OR 0.87) in nested case–control analyses [29]. Depletion of taxa, including *Neisseria elongata* and several *Streptococcus* species (*S. mitis*, *S. salivarius*, *S. thermophilus*, and *S. australis*), has also been reported in PDAC cohorts [19,24,27,29].

Among specific periodontal pathogens, *Porphyromonas gingivalis* has been most consistently associated with pancreatic cancer risk, with serum IgG levels an independent risk factor for orodigestive cancer mortality separate from periodontal disease [31]. Elevated antibody titres against *P. gingivalis* have been linked to approximately a two-fold increased risk of pancreatic cancer [30], and detection of *P. gingivalis* and the facultative *Aggregatibacter actinomycetemcomitans* in oral wash samples has been associated with increased pancreatic cancer risk in prospective analyses [29]. However, findings vary according to sampling strategy and taxonomic resolution. For example, tongue-coating analyses reported reduced abundance of *Porphyromonas* spp. in pancreatic head cancer compared with controls, alongside increased *Leptotrichia* and *Fusobacterium* [26], noting that genus-level classification does not distinguish *P. gingivalis* specifically.

Mechanistic data provide biological plausibility for these associations in pre-clinical models. In murine models, administration of *P. gingivalis* by oral lavage resulted in oral-to-pancreatic translocation and an altered pancreatic tumour microbiome composition. Repeated exposure accelerated the progression of pancreatic intraepithelial neoplasia to pancreatic cancer. Specific effects of *P. gingivalis* on acinar cells and pancreatic cancer cell lines were studied in vitro, and infection-induced acinar-to-ductal metaplasia markers (SOX9 and CK19) and intracellular bacteria protected pancreatic cancer cells from reactive oxygen species-mediated cell death [32]. While these findings support a potential contributory role, translation to human disease requires further confirmation.

High serum antibody titres against commensal oral bacteria have been associated with a 45% lower overall pancreatic cancer risk compared with low titres [30], suggesting a health-associated oral microbiota in these cohorts, rather than a dysbiotic one, containing *P. gingivalis* and *Fusobacterium.*

### 2.2. Tumour Microbiome

Evidence indicates that microbial colonisation of pancreatic tumours contributes to pancreatic cancer biology and immune regulation. Endoluminal gut bacteria have been shown to migrate into the pancreatic tumour microenvironment. Using fluorescently labelled *Enterococcus faecalis* and *Escherichia coli*, Pushalkar et al. [16] demonstrated bacterial translocation into pancreatic tumours in murine models. Both murine and human studies report a higher bacterial burden in pancreatic cancer tissue compared with normal pancreas [16,33].

Sequencing analyses demonstrate compositional differences between tumour and adjacent pancreatic tissue. Luo et al. [34] identified significant differences in alpha- and beta-diversity, with enrichment of the order Pseudomonadales, the Pseudomonadaceae genus and *Pseudomonas* species within tumour samples. Members of the class Gammaproteobacteria account for a substantial proportion of the intratumoural bacterial load, and Geller et al. [33] reported that 86/113 (76%) of pancreatic tumours were positive for Gammaproteobacteria. Additional taxa enriched within tumour tissue include *Lactobacillus* spp., *Akkermansia muciniphila*, and *Bacteroides* spp. relative to the adjacent non-tumour pancreas (Kartal et al., 2022 [35]). Ghaddar et al. [36] detected cell-associated bacteria in approximately 44% of tumour samples, but not in normal pancreatic tissue, including *Campylobacter* spp., *Fusobacterium nucleatum*, *Leptotrichia* spp., and *Clostridioides difficile*. Collectively, these findings support the presence of a distinct intratumoural microbial community in pancreatic cancer (Figure 1).

Functional studies suggest that these microbial communities influence the tumour immune microenvironment. In murine models, antibiotic-mediated microbial depletion resulted in increased intratumoural T-cell infiltration, reduced myeloid-derived suppressor cells (MDSCs), and a shift in tumour-associated macrophage (TAM) polarisation towards an M1 phenotype [16]. Chemokine profiling demonstrated increased expression of M1-associated chemokines, and whole-pancreas transcriptional analysis showed upregulation of genes involved in T-cell proliferation and immune activation. CD4^+^ and CD8^+^ intratumoural T cells exhibited increased PD-1 expression following microbial depletion, consistent with remodelling of immune tone [16].

Transcriptomic integration further links intratumoural bacteria with oncogenic and immune signalling pathways. Ghaddar et al. [36] associated microbial presence with pathways related to extracellular matrix interaction, cell motility, MET–PTK2 signalling, and PD-1 signalling. T-cells within these tumours demonstrated altered activation signatures, including upregulation of PD-1 signalling and intracellular infection response pathways, alongside downregulation of FOXO-mediated transcription and interferon-γ signalling. While these data demonstrate strong associations between microbial composition and immune architecture, causality remains to be fully defined.

Emerging data further suggest that oncogenic mutations may shape the intratumoural microbial niche. KRAS-mutant tumours demonstrate distinct microbial profiles, and experimental models indicate that KRAS G12D signalling alters epithelial permeability and facilitates microbial invasion, establishing bidirectional crosstalk between oncogenic pathways and tumour-associated bacteria [37]. These findings support a model in which microbial composition is not merely permissive but may be co-evolved with tumour genotype.

Intratumoural microbial composition has also been linked to chemotherapy response and survival outcomes [38,39]. In intestinal microbiota-colonised murine models, chemotherapy-responsive tumours exhibited increased CD8^+^ T-cell infiltration and reduced neutrophil frequencies compared with non-responsive tumours [39]. Riquelme et al. [38] reported that long-term pancreatic cancer survivors (median 9.66 years) had high intratumoural microbial alpha diversity with a unique intra-tumoural microbiome signature, markedly different when compared to short-term pancreatic cancer survivors (median 1.66 years) whose tumour microbiome had low diversity. The microbial signature comprised *Pseudoxanthomonas*, *Streptomyces*, *Saccharopolyspora*, and *Bacillus clausii* and was present in both discovery and validation cohorts, with positive correlations between microbial diversity and CD8^+^ T-cell and granzyme B-positive cell densities. Species-level composition paired with a diversity-based metric may be more informative than either of these alone.

Metabolic pathway analyses suggest that tumour-associated microbiota may influence tumour metabolic reprogramming. Luo et al. [34] reported upregulation of amino acid metabolic pathways, including arginine and proline metabolism and arginine biosynthesis in pancreatic cancer tissue relative to the adjacent pancreas. Although these findings indicate a potential link between microbial composition and tumour metabolism, mechanistic relationships remain incompletely defined.

Intratumoural bacteria may also directly influence chemotherapeutic efficacy. Geller et al. [33] demonstrated that certain Gammaproteobacteria express a long isoform of cytidine deaminase capable of metabolising gemcitabine (2′,2′-difluorodeoxycytidine) to its inactive metabolite, 2′,2′-difluorodeoxyuridine. In murine models, antibiotic administration restored gemcitabine sensitivity in colonised tumours. Given the high prevalence of Gammaproteobacteria in pancreatic cancer samples (76%), these findings provide a mechanistic basis for microbiome-associated chemoresistance, although translation to clinical practice requires further investigation.

### 2.3. Biliary Microbiome

Recent studies indicate that the biliary microbiome is altered in pancreatic cancer and differs from that observed in benign pancreatic disease. Using 16S rRNA sequencing, multiple investigations have identified dysbiosis characterised by altered microbial diversity and enrichment of specific taxa in pancreatic cancer-associated bile samples. Genera reported to be enriched include *Neisseria*, *Sphingomonas*, *Caulobacter*, *Dickeya*, the *hallii* group, *Bacteroides*, *Faecalibacterium*, *Escherichia–Shigella*, *Ruminococcus*, and *Rothia*, with Proteobacteria frequently representing the dominant phylum (Figure 1) [40,41,42,43].

Alpha diversity appears to remain unchanged in the biliary microbiome; however, beta-diversity analyses consistently demonstrate tighter clustering, suggesting high bacterial intra-group similarity with a disease-specific microbial signature [40,41,42]. Jiang et al. [42] reported that enrichment of Actinomycota in bile was associated with longer progression-free survival (PFS), whereas Bacillota enrichment correlated with shorter PFS. Although these findings suggest potential prognostic relevance, validation in larger cohorts is required.

Non-bacterial members of the biliary microbiome may also contribute to dysbiosis. Increased detection of fungal DNA, including *Candida* species, has been reported in bile samples from pancreatic cancer patients [43,44], extending the concept of the biliary microbiome beyond bacterial taxa.

Instrumentation has a substantial impact on the microbial composition. Preoperative biliary drainage and stenting are consistently associated with increased bacterial load and altered microbial profiles [45,46,47,48]. Experimental data suggest that sterile bile exerts anti-tumour effects, attributed to properties of conjugated bile acids. In murine and in vitro models, bile from stented patients or bile pre-incubated with live *Enterococcus faecalis* or *Streptococcus oralis* attenuated these antitumour effects [46]. Functional analyses further suggest that biliary microbial alterations may influence metabolic pathways relevant to tumour biology, including bile acid and fatty acid metabolism.

Neoadjuvant therapy and biliary stenting have been associated with increased abundance of *Enterococcus* and *Klebsiella* species in bile from patients undergoing pancreaticoduodenectomy, with many isolates demonstrating resistance to cephalosporins [48]. These findings have implications for perioperative antibiotic strategies. However, pre-operative biliary drainage itself has not been found to influence pancreatic cancer outcomes or survival other than an increase in surgical site infections [49,50,51]. Retrospective analyses have also suggested that colonisation with Gammaproteobacteria or *Klebsiella pneumoniae* in bile may influence outcomes in patients receiving gemcitabine-based chemotherapy, with reported associations with overall survival (OS) and PFS [47,52]. Stenting was significantly associated with an increase in gammaproteobacteria but again was not itself an independent risk factor for alterations in overall survival [47]. However, these studies are culture-based and retrospective, and causal relationships remain to be established.

### 2.4. Gut Microbiome

A role for the gut microbiome in pancreatic cancer is being increasingly recognised. In healthy individuals, the gut microbiota is predominantly composed of Firmicutes and Bacteroidetes [53]. Multiple studies employing 16S rRNA and shotgun metagenomic sequencing have reported reduced alpha diversity and species evenness in the faecal microbiota of patients with PDAC compared with healthy controls [16,19,35,54,55].

Across cohorts, enrichment of Proteobacteria, including *Veillonella*, *Klebsiella pneumoniae* and members of the Enterobacteriaceae family, has been observed, alongside depletion of Firmicutes and short-chain fatty acid-producing taxa such as *Bifidobacterium bifidum* [19,35,54,55]. These findings are consistent with a shift towards a pro-inflammatory microbial profile, although associations remain observational. Increased abundance of oral-associated taxa within stool samples from pancreatic cancer patients has also been reported, supporting cross-niche microbial overlap (Figure 1) [19,35]. The gut microbiome, however, is not limited to bacterial species alone.

Emerging evidence indicates that dysbiosis in pancreatic cancer extends to the gut virome. Integrated analysis of two European cohorts identified 219 viral operational taxonomic units differentially abundant in pancreatic cancer, with reduced virome diversity correlating with advancing disease stage [31]. Enriched phages were predicted to infect *Veillonella*, *Enterobacter*, *Fusobacterium nucleatum* and *Escherichia coli*; taxa independently reported as expanded in pancreatic cancer, whereas depleted viral signatures targeted short-chain fatty acid-producing commensals, including *Faecalibacterium prausnitzii* and *Ruminococcus* [31]. These parallel shifts suggest coordinated virus–bacteria network restructuring rather than isolated bacterial change. Virome-based classifiers achieved discriminatory performance comparable to bacterial models (AUC up to 0.88), including in early-stage disease, supporting the relevance of viral alterations within a broader, dysbiotic landscape.

Gut microbial composition is influenced by established pancreatic cancer risk factors and disease-related physiological changes. Smoking, a recognised risk factor for pancreatic cancer, alters gut microbial structure; cessation has been associated with increased abundance of Firmicutes and Actinobacteria, reduced Bacteroidetes and Proteobacteria, and increased microbial diversity [56]. Biliary obstruction in pancreatic cancer is also associated with altered gut microbial profiles, including increased abundance of *Streptococcus* species, potentially mediated by changes in bile acid composition [19,55]. Altered bile acid signalling may contribute to gut dysbiosis and impaired mucosal barrier integrity [57]. Additional studies have reported associations between gut microbial composition and tumour localisation (head versus body/tail), systemic inflammation, and liver dysfunction markers [13,58]

Microbial composition has also been linked to therapeutic response. Enrichment of *Bacteroides fragilis* and *Bacteroides thetaiotaomicron* has been observed in patients responding to chemotherapy [39]. Long-term survivors have demonstrated increased abundance of *Faecalibacterium prausnitzii* and *Akkermansia muciniphila*, taxa implicated in immune modulation [38]. However, causality remains to be established, and inter-cohort variability persists. Mechanistically, the gut microbiota plays a central role in mucosal immune homeostasis. Interactions between microbial communities and mucosal T and B cells contribute to barrier integrity and immune regulation. Dysbiosis may disrupt this balance, increasing permeability and promoting proinflammatory signalling [59]. Elucidating the mechanisms distinguishing homeostatic from pathogenic host–microbe interactions may identify therapeutic targets and inform strategies to enhance cancer treatment efficacy. Indeed, most studies detailing dysbiosis [35,54,55] and the presence of specific taxa as risk factors for pancreatic cancer have been performed in treatment-naïve cohorts. Given that systemic chemotherapy can independently reshape gut microbial diversity, its influence must be considered when interpreting dysbiosis patterns, particularly as prospective studies increasingly examine microbiome-treatment response associations.

### 2.5. Holobiont-Level Biological Signals

**Figure 2:**
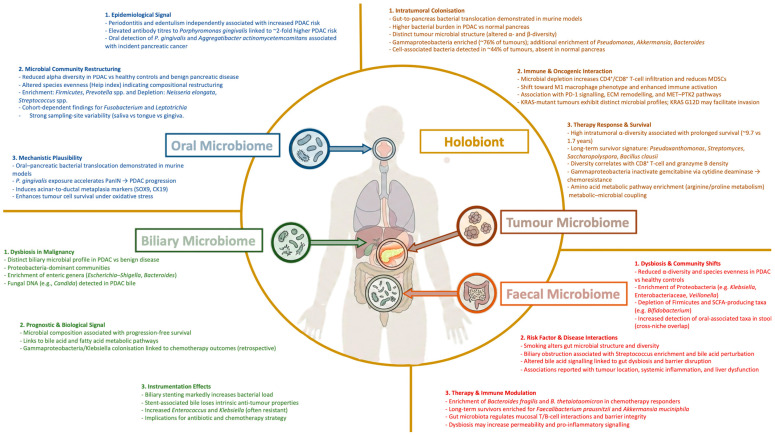
A Conceptual model of oral, gut, biliary and intratumoural microbiomes within a holobiont framework in pancreatic cancer.

Comparative analyses across anatomical niches demonstrate interconnection between the oral, intestinal, and pancreatic microbiomes in pancreatic cancer (Figure 2). Significant differences in bacterial taxa have been observed across oral, faecal, and pancreatic tissue samples, and paired salivary–faecal metagenomic analyses suggest that a proportion of pancreatic cancer-associated gut microbes may originate from the oral cavity [19,28,35]. Increased oral-intestinal strain transmission has been reported in pancreatic cancer, including co-abundance patterns involving taxa such as *Fusobacterium nucleatum* subsp. *Vincentii* and *Gemella morbillorum* across body sites [28,35]. These findings support a multi-site host–microbe framework in pancreatic cancer and emphasise the importance of integrated sampling approaches when investigating pancreatic cancer biology.

However, a holobiont perspective extends beyond the presence or absence of microbial taxa. Holobionts function as metabolically integrated systems in which microbial functional capacity, including metabolite production (e.g., short-chain fatty acids, indoles, polyamines), quorum-sensing signals, and biochemically modified host-derived molecules, play a central role in shaping immune tone and tumour biology. Microbial metabolites act as biochemical messengers that influence host physiology, epithelial barrier function, and immune programming, illustrating that functional signalling networks, rather than taxonomy alone, maintain holobiont homeostasis [10].

Emerging “holo-omics” approaches highlight that host–microbe interactions must be interpreted through joint functional pathways, integrating metagenomics with transcriptomics, metabolomics, and immunophenotyping to resolve how microbial metabolic activity contributes to ecological stability, dysbiosis, or tumour-associated immune reprogramming [60,61].

Thus, a true holobiont framework emphasises that functional microbial capacities, metabolic, immunomodulatory, and signalling, are the major drivers of cross-site microbiome connectivity in pancreatic cancer, underscoring the importance of integrated, multiomic sampling strategies when investigating disease biology.

The microbiome is also being explored as a diagnostic tool. Although microbial variation shows modest associations with clinical variables such as age, diabetes, and jaundice, faecal microbiome composition remains independently associated with pancreatic cancer status after adjustment for confounders [35]. Several taxa are recurrently reported as altered in pancreatic cancer, including enrichment of oral bacteria *Veillonella atypica*, *Fusobacterium nucleatum/hwasookii*, and *Alloscardovia omnicolens*, throughout the holobiont alongside depletion of taxa such as *Faecalibacterium prausnitzii*, *Bifidobacterium bifidum*, *Bacteroides coprocola*, and *Romboutsia timonensis* within the gut.

Machine-learning classifiers derived from faecal metagenomic classifiers show good diagnostic performance for pancreatic cancer (AUROC ≈ 0.84), with sensitivities of ~50–60% at 90% specificity, improving to ~80–85% sensitivity (AUROC ≈ 0.94) when combined with CA19-9 [35]. Similarly, integration of oral and faecal microbiome profiles with serum markers (CA19-9, CEA, Dupan-2, Span-1) has achieved AUC values of 0.81–0.97, with sensitivities and specificities generally ranging between ~70–90%, demonstrating improved accuracy compared to individual biomarkers alone [19]. These findings suggest that microbiome profiling may complement existing diagnostic strategies; however, population heterogeneity, methodological variability, and the need for external validation remain important limitations.

## 3. Interventions

### 3.1. Oral Interventions

Within the holobiont framework, the oral microbiome constitutes a structured ecological niche that interacts with distal microbial communities and systemic immunity [12]. Oral dysbiosis reflects ecological imbalance rather than isolated pathogen overgrowth and may influence systemic inflammatory tone through transient bacteraemia, cytokine signalling and immune activation [62]. Periodontal intervention studies demonstrate that the oral microbiome is modifiable, with treatment capable of reshaping host–microbial immune networks and reducing systemic inflammatory signatures [63]. Potential modulation strategies include mechanical plaque disruption, targeted periodontal therapy, ecological probiotic or prebiotic approaches, and lifestyle modification affecting oral metabolic function. Contemporary ecological models emphasise restoration of symbiosis through environmental niche manipulation rather than microbial eradication [12]. Emerging approaches such as oral microbiome transplantation (OMT) are now being explored as novel strategies to restore oral ecological balance. Early experimental and pre-clinical work and in vitro models suggest OMT may reduce inflammation, inhibit pathogenic biofilms, and re-establish a stable oral microbiota, although human clinical evidence is still lacking [64,65].

However, evidence in pancreatic cancer remains indirect and no trials currently exist that directly investigate the modulation of the oral microbiome to influence outcomes. Prospective trials integrating periodontal interventions to manipulate holobiont composition are required to determine whether they could produce an impact on systemic or tumour-associated microbial signatures and subsequent outcomes.

### 3.2. Dietary Changes

Diet-microbiome interactions are increasingly recognised as relevant in multiple disease contexts, including pancreatic cancer and therapy response [66,67]. Western dietary patterns, characterised by low fibre intake and high convenience food consumption, have long been associated with adverse metabolic and inflammatory profiles [68]. Of particular interest are short-chain fatty acids and tryptophan-derived metabolites, which influence mucosal immunity, gut barrier integrity, and inflammatory signalling, whilst increased dietary fibre intake promotes expansion of short-chain fatty acid-producing taxa and elevates short-chain fatty acid concentrations [39,67,68]. As illustrated in Figure 1, bacteria associated with improved outcomes in pancreatic cancer, predominantly utilise substrates derived from fibre, resistant starch, fermented foods, and polyphenols. In contrast, diets high in simple carbohydrates and certain protein sources tend to favour microbial populations broadly linked to poorer outcomes. Dysbiosis, characterised by reduced alpha diversity, reflects a loss of microbial heterogeneity, underscoring the critical role of maintaining a varied and diverse diet. Symbiosis between humans and bacteria has been developed gradually over a period of millennia and as with any evolutionary characteristic, will have been selectively developed to benefit the survival of both [69]. Any rapid alteration in external factors such as key food sources, demonstrated in the Western diet by reduced fibre and greater consumption of processed foods, simple carbohydrates and protein, will disrupt the holobiont, causing imbalance, just as it would in any other well-established ecosystem.

Dietary modification can induce rapid alterations in gut microbial composition, with measurable shifts occurring within days [39,70]. In pancreatic cancer, a tryptophan-rich diet increased microbial production of indole-3-acetic acid (3-IAA) within four days. During chemotherapy, 3-IAA is oxidised by myeloperoxidase, leading to reduced expression of reactive oxygen species-detoxifying enzymes, reactive oxygen species accumulation, and impaired tumour-cell metabolic fitness and proliferation. In human pancreatic cancer cohorts and gnotobiotic mouse models, higher circulating 3-IAA levels were associated with improved chemotherapy efficacy. *Bacteroides fragilis* and *Bacteroides thetaiotaomicron* were identified as 3-IAA-producing species in vitro, and treatment responsiveness correlated with increased CD8^+^ T-cell infiltration and reduced neutrophil abundance [39]

Short-term dietary interventions comparing plant-based and animal-based diets demonstrate substantial microbial and metabolic shifts within five days, including expansion of bile-tolerant taxa (*Alistipes*, *Bilophila*, and *Bacteroides*) and reduction in fibre-fermenting Firmicutes (*Roseburia*, *Eubacterium rectale*, and *Ruminococcus bromii*), accompanied by altered short-chain fatty acids and bile-acid metabolism [70].

However, the durability and therapeutic relevance of dietary microbiome modulation remain uncertain. Current evidence is limited by small study cohort sizes and a lack of long-term interventional studies, and diet cannot yet be recommended as a microbiome-targeted therapeutic strategy [71]. Longer interventions, including Mediterranean-style or plant-based diets over three months, produce modest changes in microbial abundance and inflammatory correlations without substantial shifts in overall diversity [72]. Combined caloric restriction and exercise programmes also modify microbial composition and metabolic parameters, including increased abundance of *Akkermansia muciniphila* and correlations between microbial shifts, short-chain fatty acids, and body-composition improvements [73]. Mechanistic studies further suggest that nutrient availability and ecological niche competition determine bacterial strain engraftment, underscoring how dietary substrates shape colonisation dynamics [74]. Conversely, the functional gene repertoire of the microbiota dictates which microbial enzymes are expressed, thereby determining the spectrum of metabolites produced, including short-chain fatty acids, indoles, polyamines, and bacterially modified host-derived molecules, each of which can exert immunomodulatory, metabolic, or barrier-regulatory effects within the holobiont.

Collectively, diet plays a significant role in shaping microbial composition, metabolite production, and immune signalling, and may influence treatment response through modulation of the bacterial holobiont. Substantial evidence demonstrates that dietary factors directly impact the gastrointestinal microbiome. Given the well-established crosstalk between microbial niches, these changes may extend to the biliary and tumour-associated microbiomes [38]. Furthermore, alterations in both the tumour and biliary microbiome have been shown to influence chemotherapy outcomes in pancreatic cancer. Nonetheless, robust longitudinal clinical studies are required before dietary modulation can be incorporated into therapeutic strategies in pancreatic cancer.

### 3.3. Probiotics, Prebiotics and Immunonutrition

Probiotics are defined as “live microorganisms that, when administered in adequate amounts, confer a health benefit on the host” [75] and their use in oncology is increasing. In pancreatic cancer, probiotic supplementation during palliative chemotherapy was associated with improved overall survival compared with controls (median 12 months [9,10,11,12,13,14,15,16,17,18,19] vs. 10 months [9,10,11]; *p* = 0.026) [76]. In the perioperative setting, a randomised controlled trial demonstrated that probiotic administration (*Enterococcus faecalis* T-110, *Clostridium butyricum* TO-A, *Bacillus mesentericus* TO-A) significantly reduced infectious complications following pancreaticoduodenectomy [77]. In other malignancies, *Lactobacillus* and *Bifidobacterium* species have been associated with supportive roles in cancer prevention and therapy [78].

Oral immunonutrition, typically comprising arginine, omega-3 fatty acids and nucleotides, with or without glutamine, has demonstrated consistent reductions in postoperative infectious complications and hospital length of stay in gastrointestinal cancer surgery, including colorectal cancer and pancreatic cancer [79,80]. Meta-analyses confirm reductions in infectious morbidity and hospital stay, although effects on overall complications and mortality are less clear [79,81]. In colorectal cancer, immunonutrition has been associated with increased intra-tumoural lymphocyte infiltration; this effect has not yet been demonstrated in pancreatic cancer [81].

In pancreatic surgery, immunonutrition is recommended within ERAS^®^ pathways and supported by meta-analyses showing reduced infectious complications and length of stay, but with no clear mortality benefit [82,83]. Optimal formulation, timing and the addition of synbiotics (a combination of pro and prebiotics) sustained omega-3 impregnation or vitamin D, as investigated in the SIO3D trial, remain under evaluation (Table 1). Overall, probiotics and immunonutrition appear to reduce infectious morbidity in pancreatic cancer care, particularly in the perioperative setting. However, effects on oncological outcomes and superiority over standard nutritional support require further high-quality evidence.

### 3.4. Faecal Microbiota Transplantation (FMT)

Faecal microbiota transplantation (FMT) has emerged as a potential strategy to modulate the holobiont in pancreatic cancer. In a landmark study examining tumour microbiome composition in relation to survival, long-term PDAC survivors demonstrated significantly greater intratumoural microbial diversity compared with short-term survivors, accompanied by increased CD8+ T cell infiltration. Stool from human long-term pancreatic cancer survivors, short-term survivors or healthy controls was transferred to mice with pancreatic cancer. The mice that received stool from the long-term survivors demonstrated differentially altered tumour microbiomes and reduced tumour growth. The FMT from long-term survivors into the mice reshaped the tumour microbiome, enhanced antitumour immune activation, and reduced tumour growth [38]. Further research supports this, demonstrating that intratumoural microbiota in pancreatic cancer predominantly originate from the oral and gut microbiomes. This dynamic interplay allows for modulation of the tumour microbiome through FMT alongside immunomodulatory effects [51]

Mechanistically, FMT has been associated with modulation of innate and adaptive immune pathways. Reduction in Toll-like receptor 4 signalling and increased expression of mucin and tight junction proteins may improve gut barrier integrity and reduce inflammation [84,85]. Microbiota-derived inosine has been shown to promote macrophage differentiation, naïve CD8^+^ T-cell activation, and T helper 1 responses via adenosine 2A receptor signalling [86]. Microbial modulation has also been linked to altered PD-1 pathway activity, relevant to immune checkpoint responsiveness in pancreatic cancer [16,36,87].

Clinical evidence remains in the early phase. A phase I trial (NCT06393400) evaluating oral FMT capsules combined with gemcitabine and nab-paclitaxel in advanced pancreatic cancer reported acceptable safety in the first ten patients, with one partial response and four cases of stable disease among five evaluable participants; CA19-9 reduction > 50% was observed in five of seven patients [88]. A second phase I study (NCT04975217) is investigating perioperative FMT delivered initially via colonoscopy, followed by capsule therapy (Table 1). Although exciting, the current data are preliminary. Larger, controlled studies are required to determine whether FMT produces durable immune modulation or a clinically meaningful survival benefit in pancreatic cancer. Importantly, existing FMT studies do not yet adopt a true holobiont perspective, as they typically focus on FMT composition or clinical endpoints without integrating multi-site microbial ecology, functional metabolic capacity, or cross-compartment host–microbe signalling. The benefit of FMT to the holobiont is best demonstrated by the improvement in many extra-intestinal diseases and malignancies reaching beyond a localised response [89].

The clinical application of FMT raises important ethical considerations that warrant acknowledgement. Although its risk profile compares favourably to many conventional therapies, the long-term consequences of donor microbiome transfer remain incompletely characterised, necessitating continued post-procedure surveillance [90,91]. Rigorous donor screening is essential and patients must be fully informed of these uncertainties prior to consent.

### 3.5. Engineered Microbial Therapies

Genetically engineered bacteria are being developed as targeted anti-tumour platforms in pancreatic cancer, although these strategies remain pre-clinical. Hypoxia-resistant *Escherichia coli* have been engineered to secrete cyst(e)inase, depleting cystine and triggering ferroptosis within pancreatic tumour cells [92]. Similarly, MG1655 *E. coli* modified with CDH17 nanobodies selectively target CDH17-positive tumour cells, including pancreatic cancer. When combined with photothermal therapy, this approach enhanced macrophage infiltration and STING pathway activation, resulting in tumour eradication in murine models [93].

Oncolytic bacterial platforms have also been explored. Engineered *Salmonella* expressing the pore-forming toxin cytolysin A accumulated within tumour tissue following intravenous administration and inhibited tumour growth in both subcutaneous and orthotopic murine models [94]. Microbial components may further modulate immune signalling; bacterial lipopolysaccharide (LPS) has been shown to induce tumour PD-L1 expression [95], while modified *Lactobacillus rhamnosus* GG incorporating a gallium-polyphenol network selectively targeted pancreatic cancer tumours, reduced intratumoural Proteobacteria and attenuated microbiome-derived LPS signalling [92].

There is also emerging scope for bacteriophages within engineered microbial strategies for pancreatic cancer. Phage display technology has been used to identify peptides with high specificity for pancreatic cancer cells, enabling targeted drug delivery and diagnostic applications. Engineered phages offer modular, highly adaptable platforms capable of presenting tumour-homing ligands, delivering therapeutic payloads, and modulating immune responses. Recent microbiome-based therapeutic reviews include bacteriophages among the most promising microbial tools for PDAC, positioning phage-derived agents as a logical extension of engineered microbial therapies in this setting [96,97,98].

Collectively, these approaches illustrate the potential of engineered microbes to deliver targeted cytotoxic, metabolic or immunomodulatory effects within the pancreatic tumour microenvironment. However, all data to date derive from pre-clinical models, and translational challenges, including safety, tumour specificity and regulatory complexity, remain substantial.

### 3.6. Antibiotics

Antibiotic-mediated microbiome depletion has been shown in pre-clinical models to modulate the gut–pancreas axis. In murine pancreatic cancer, microbial ablation increased CD8^+^ T-cell infiltration, shifted tumour-associated macrophages toward an M1 phenotype, and enhanced responsiveness to PD-1 blockade [16]. These findings support the concept that microbial composition influences intratumoural immune tone and chemotherapy responsiveness.

Retrospective clinical studies have suggested a potential survival association between antibiotic exposure and gemcitabine-based chemotherapy. Quinolone use was associated with improved median overall survival (48.8 vs. 26.2 months; *p* = 0.006), although without a matched comparison, causal inference is not possible here [52]. In early-stage pancreatic cancer receiving adjuvant gemcitabine, antibiotic exposure within one month of chemotherapy initiation was associated with a 37% improvement in overall survival and 30% improvement in cancer-specific survival [99]. In metastatic disease, antibiotic use was associated with longer overall survival (13.3 vs. 9.0 months) and improved progression-free survival, particularly in patients receiving gemcitabine-based regimens [99,100]. Nevertheless, any apparent benefit must be balanced against antimicrobial-resistance (AMR) stewardship, given quinolones’ well-documented propensity to select for resistance.

Beyond systemic therapy, increasing evidence implicates the perioperative microbiome in post-resection morbidity and mortality. Multi-site 16S rRNA profiling in patients undergoing pancreaticoduodenectomy demonstrates that bile, gastric, pancreatic duct and jejunal microbiota cluster together and that *Enterococcus*-enriched bile is associated with deep or organ-space surgical site infection, ICU admission and increased 12-month mortality. Preoperative biliary instrumentation appears to promote bacterobilia, facilitating upper gastrointestinal microbial translocation and increasing postoperative infectious burden [40]. These findings suggest that perioperative microbial composition is not merely a contaminant but biologically linked to surgical outcome. Consistent with this, a recent systematic review and meta-analysis comparing broad-spectrum penicillin-based prophylaxis (e.g., piperacillin–tazobactam) with cephalosporin-based regimens in pancreatoduodenectomy demonstrated significant reductions in surgical site infection, clinically relevant POPF, organ/space infection and mortality, particularly in patients with preoperative biliary drainage [101]. The broader antimicrobial coverage of Enterococcus and *Enterobacter* species, commonly isolated from bile, may account for these differences. These data suggest that antibiotic strategy in pancreatic cancer surgery may represent a form of targeted microbiome modulation rather than simple infection prophylaxis.

However, antibiotics remain a non-selective intervention. While short-term microbial depletion or targeted perioperative coverage may reduce infectious morbidity and potentially influence tumour immune dynamics, broad-spectrum exposure carries risks of dysbiosis, antimicrobial resistance and unintended systemic consequences [102]. Current survival data derive largely from retrospective cohorts, and perioperative microbiome-directed antibiotic strategies require prospective validation (Table 1). Antibiotics, therefore, occupy a complex position within the holobiont in pancreatic cancer: capable of modulating immune and surgical outcomes, yet inherently imprecise and carrying a great risk of driving dysbiosis and antimicrobial resistance. Future approaches may therefore require microbiome-informed stratification rather than empirical broad-spectrum ablation and current consideration of antimicrobial resistance. As antimicrobial-sparing alternatives, faecal microbiota transplantation (FMT) and bacteriophage-based strategies are under active investigation [103]: systematic reviews and early trials across cancer types suggest FMT is feasible and can reprogramme gut ecosystems and tumour-immune tone (e.g., increased tumour-infiltrating lymphocytes), with signals of clinical activity alongside immunotherapy—though robust RCTs in PDAC are still needed. In parallel, engineered phage platforms and phage-display-derived tumour-homing peptides offer modular, targeted delivery and on-site antimicrobial or immunomodulatory functions within the tumour microenvironment; recent PDAC-focused and broader oncology reviews place phages among the most promising microbiome-based tools to reduce antibiotic pressure while enabling precision microbial modulation. Future approaches may therefore require microbiome-informed stratification, prioritising FMT/phage-based interventions where appropriate, over empirical broad-spectrum ablation, with explicit integration of AMR stewardship principles [8,104,105].

## 4. Conclusions

Pancreatic cancer remains a complex and challenging cancer, though it is becoming clear that it exists within a broader ecological system encompassing oral, intestinal, biliary and intratumoural microbial communities. Across these anatomical niches, compositional shifts, microbial translocation, and metabolite signalling interact with immune architecture, tumour metabolism and therapeutic responsiveness. The concept of the pancreatic holobiont can provide a framework to interpret these interactions and may help explain inter-patient heterogeneity in disease progression and treatment outcomes.

However, important distinctions must be made. Much of the current evidence remains associative, with variability across cohorts, sequencing platforms and sampling strategies. Studies and trials are small, making it difficult to detect a clinically significant difference. Diversity metrics are inconsistent predictors, and species-level resolution is often required to uncover meaningful patterns. Pre-clinical models demonstrate compelling mechanistic links between microbial composition, immune modulation and chemoresistance, but translation to clinical practice is still immature. Interventions ranging from dietary modification and probiotics to FMT illustrate therapeutic potential and represent an exciting addition to the pancreatologists’ armoury, but robust prospective trials are required to determine acceptability, safety and any true oncological benefit. It is important to remain aware of the multifactorial nature of pancreatic cancer treatment in analysing the microbiome in relation to treatment responses to prevent oversimplification of conclusions. Future progress will depend on integrated, longitudinal, multi-site sampling strategies that are able to capture the dynamic nature of microbiome interactions. Standardisation of methodology, incorporation of metabolomics and transcriptomics, and mechanistically informed interventional studies will be essential.

## Figures and Tables

**Table 1:** Proposed or active interventional clinical trials in pancreatic cancer involving microbiome modulation, both directly and as an exploratory endpoint.

Name	Number	Intervention	Status	Summary
Oral Immunonutrition with Synbiotics, Omega-3 and Vitamin D in Patients Undergoing Duodenopancreatectomy for Tumoral Lesions (SIO3D)	NCT05271344	Synbioimmunotrition (combined with omega-3 and vitamin D)	R	Prospective placebo-controlled double-blinded RCT to compare standard preoperative immunonutrition with combined synbioimmunonutrition, omega-3 and vitamin D in the ability to reduce overall morbidity.
Effects of Peptamen 1.6 in Malnourished Patients (or at Risk) With Pancreatic Neoplasia Undergoing Cephalic Pancreaticoduodenectomy (CPD): A Mechanistic Study	NCT06852014	Nutrition (Peptamen 1.6)	R	Prospective, double-blinded, RCT with an in vivo component assessing the impact of Peptamen 1.6 on digestive tolerance, amino acid absorption, nutritional status, metabolic profile, inflammatory markers, and gut microbiota composition, and a mechanistic in vitro (human intestinal organoid models) component, focusing on microbiota interactions.
Pancreaze (Pancrelipase) for Patients with Pancreatic Adenocarcinoma with Cachexia and Exocrine Pancreatic Insufficiency (PANCAX-3)	NCT04098237	PERT (Pancrelipase)	ANR	Open-label single-centre prospective study of Pancrelipase in addition to standard of care in advanced pancreatic cancer patients with pancreatic insufficiency. Monitoring response with change in microbiome, activity, sleep and heart rate.
The Effect of Probiotics ATG-F4 in Cancer Patients	NCT06436976	Probiotic (Lactobacillus reuteri ATG-F4)	R	Open label single centre prospective study to assess the impact of probiotics on advanced colorectal cancer or pancreatic cancer patients receiving oxaliplatin-based chemotherapy, looking at OS, PFS, chemotherapy tolerability, inflammatory markers, weight and quality of life.
Clinical Study on Faecal Microbiota Transplantation for Diarrhoea After Total Pancreatectomy (FMT-TP)	NCT06960122	FMT (oral capsules)	NYR	Single-centre clinical trial to evaluate the efficacy and safety of FMT in alleviating post-TP diarrhoea through clinical indicators and 16S rDNA sequencing.
Chemotherapy and Stool Transplant in PDAC (CHASe-PDAC)	NCT06393400	FMT (oral capsules)	R	Phase one single-arm study aiming to look at combined therapy, assess clinical outcomes, perform gut microbiome analysis, systemic immune profiling, and explore patient-related outcomes.
Faecal Microbial Transplants for the Treatment of Pancreatic Cancer	NCT04975217	FMT (single colonoscopy delivered dose and oral capsules)	R	Pilot study to assess the safety, tolerability, and feasibility of FMT in resectable patients with PDAC and to assess any alterations in the oral, faecal and tumour microbiome and immunological changes.
Modulation of the Gut Microbiome with Pembrolizumab Following Chemotherapy in Resectable Pancreatic Cancer	NCT05462496	Antibiotics and PD-1 antagonist (Pembrolizumab)	R	Multi-institutional, single-arm pilot study of antibiotics and pembrolizumab, following chemotherapy pre-operatively for the treatment of surgically resectable pancreatic cancer, reviewing microbiome, immune impact and tumour response.
* CD318-targeted CAR-T Cell Therapy in Patients with Pancreatic Cancer (ResCPa)	NCT07153289	CAR-T Cell Therapy (CD318)	NYR	Single experimental cohort study with a Phase I dose escalation (single infusion) followed by a Phase IIa expansion cohort at RP2D with dual dosing to assess response, OS and PFS. Exploratory faecal and tumour microbiome analysis.
* TTFields and Chemotherapy in Metastatic Pancreatic Adenocarcinoma (mPANCREATIC CANCER)	NCT07284277	TTFields	R	Multi-centre phase Ib-II, non-randomised, open-label study of TTFields, concomitant with modified FOLFIRINOX for front-line treatment of metastatic PDAC, assessing response, OS and PFS. Exploratory faecal and tumour microbiome analysis.
* Neo-adjuvant Chemo-Immunotherapy in Pancreatic Cancer (NEO-IMPACT)	NCT06094140	PD-L1 antagonist (durvalumab)	ANR	Pilot study to evaluate the feasibility and safety of combining modified FOLFIRINOX with durvalumab in patients with resectable or borderline resectable PDAC, assessing response, OS and PFS. Exploratory analysis of the oral, tumour and faecal microbiome

The listed trials represent proposed (NYR, not yet recruiting; R, recruiting; ANR, active not recruiting) interventional trials on ClinicalTrials.gov as of 14 February 2025, using the search terms ‘pancreatic cancer’, ‘pancreatic neoplasms’, ‘microbiome’ and ‘microbiota’. Those that were interventional, but whose mechanism does not target the microbiome, are marked with *. FMT, faecal microbiota transplant; FOLFIRINOX, folinic acid, 5-fluorouracil, irinotecan, oxaliplatin; OS, overall survival; PFS, progression-free survival; PDAC, pancreatic ductal adenocarcinoma; PFS, progression-free survival; RCT, randomised control trial; TTFields, tumour treating fields.

## Data Availability

No new data were created or analysed in this study.
